# Utilizing real-time contrast medium to detect the fistula of giant spinal arachnoid cyst and treat with minimal invasive surgery

**DOI:** 10.1186/s12893-019-0475-y

**Published:** 2019-01-21

**Authors:** Guang-Yu Ying, Kai-Sheng Chang, Ya-Juan Tang, Chun-Yuan Cheng, Yong-Jian Zhu, Chien-Min Chen

**Affiliations:** 1 Department of Neurosurgery, Second Affiliate Hospital Zhejiang University School of Medicine, Zhejiang, China; 2 Department of Neurosurgery, Division of Neurosurgery, Department of Surgery, Changhua Christian Hospital, 135 Nanhsiao Street, Changhua, 500 Taiwan, Republic of China; 3 School of Medicine, Kaohsiung Medical University, Kaohsiung, Taiwan; 4 College of Nursing and Health Sciences, Da-Yeh University, Changhua, Taiwan

**Keywords:** Digital subtraction angiography, Cerebrospinal fluid fistula, Giant spinal arachnoid cyst, Minimally invasive surgery

## Abstract

**Background:**

Spinal arachnoid cysts are rare and have varied clinical manifestations depending on the affected spinal region and nerve roots. A complete cyst excision with fistula closure is the first choice of treatment. However, it might be difficult to localize the specific position of the fistula because previous images have no enhancements or the fistula is too tiny to be detected.

**Case presentation:**

This case is a giant lumbar extradural arachnoid cyst. We administered a lumbar injection with contrast medium into subarachnoid space under digital subtraction angiography (DSA) and disclosed the fistula. Confirming the location of fistula enabled us to perform minimally invasive surgery to ligate the fistula. Surgical intervention for a spinal arachnoid cyst might encounter the problem of the formation of a postoperative cerebrospinal fluid (CSF) fistula. We propose the option of detecting the fistula preoperatively for minimal invasive surgery. Recurrence depends on the long-term follow-up, and more cases are needed to further evaluate our technique.

**Conclusions:**

The real-time contrast medium technique for spinal arachnoid cysts contributes to the complete ligation with minimally invasive surgery.

## Background

Spinal arachnoid cysts are uncommon. They have different clinical manifestations depending on the affected spinal region and nerve roots. They are categorized as intradural or extradural lesions. Surgical intervention aims at complete cyst excision with fistula closure. However, localizing the fistula is the key to decreasing the recurrence. We present a case where the position of the fistula was detected by simultaneously performing a myelography and a digital subtraction cystography.

## Case presentation

**Fig. 1:**
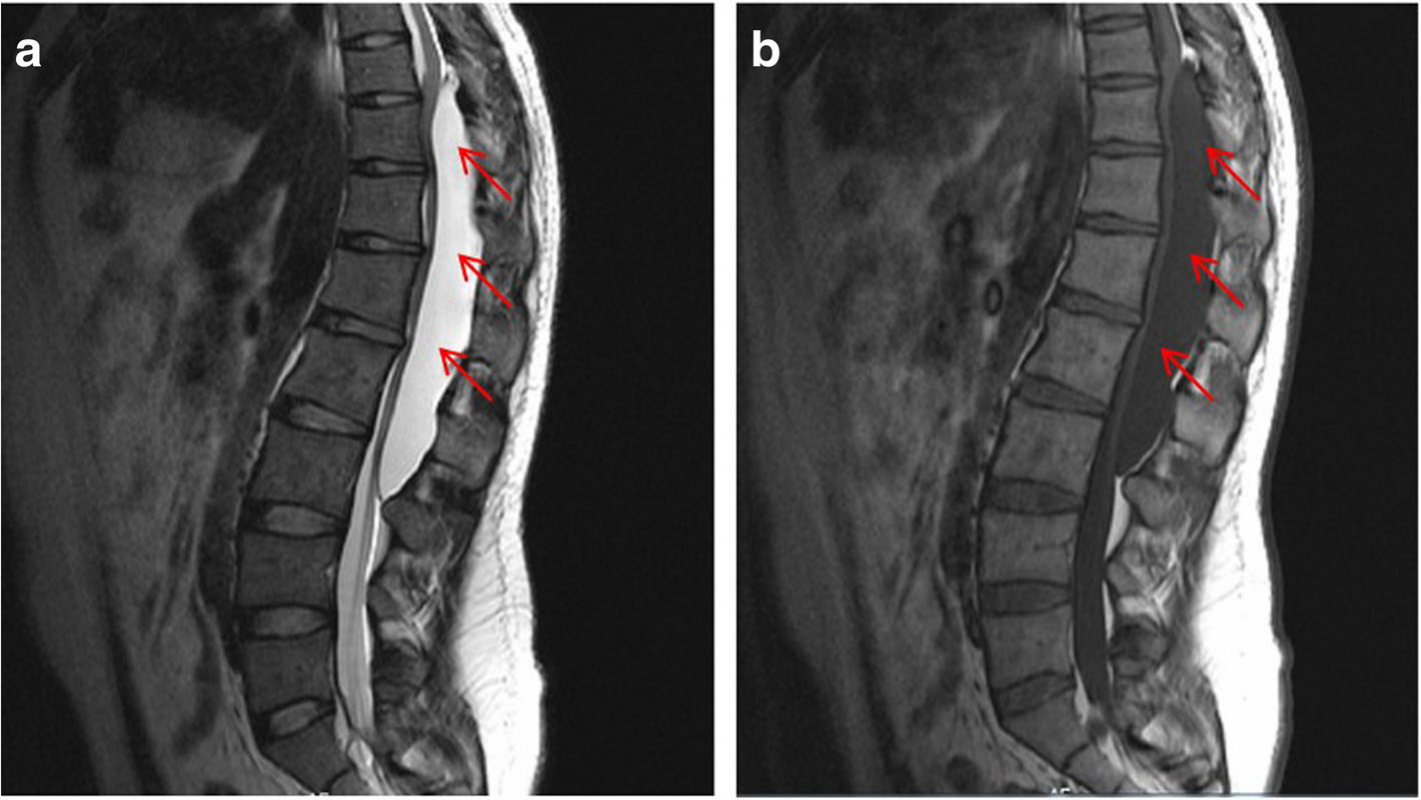
Magnetic resonance imaging revealed an intraspinal extradural tumor over T10 to L3 (red arrows), **a** Sagittal T2-weighted **b** T1-weighted

**Fig. 2:**
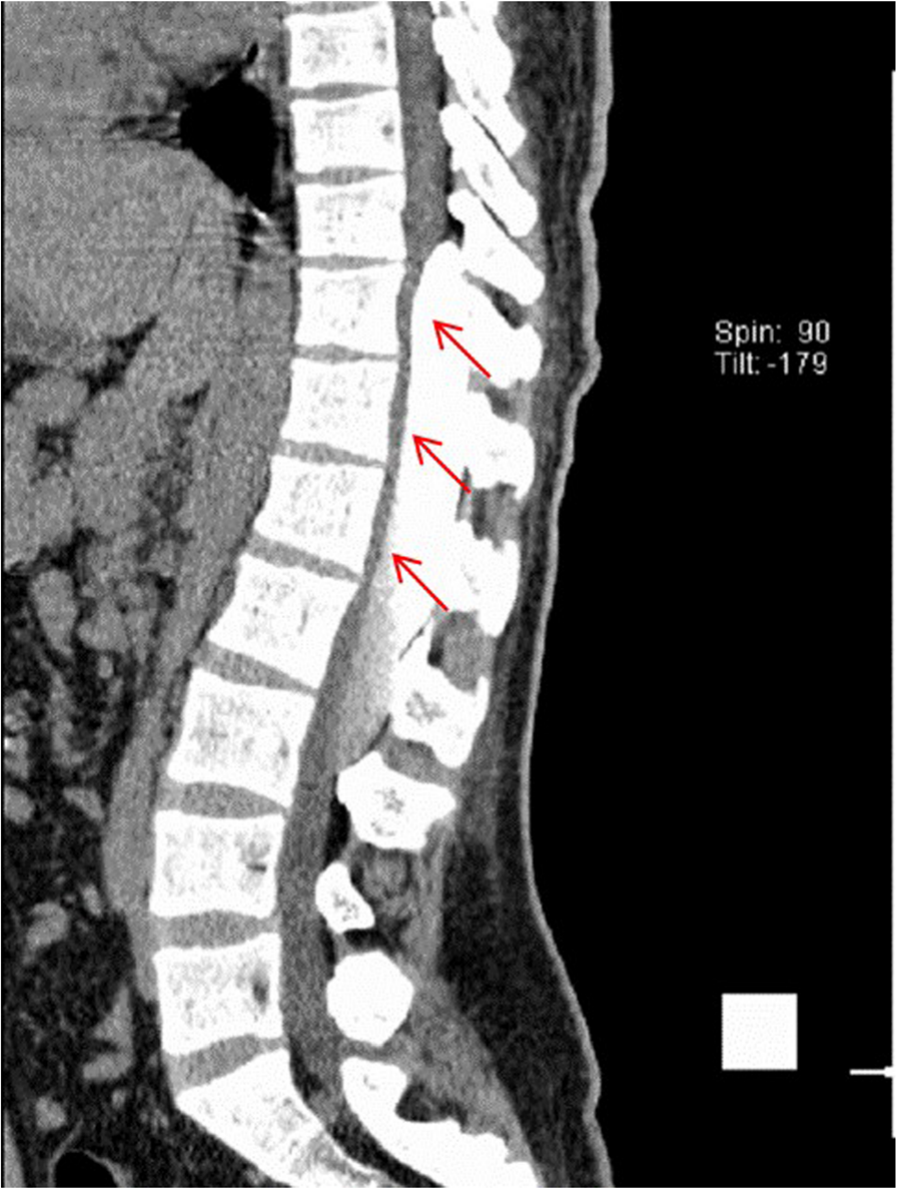
Cystography: contrast medium was injected one hour before (red arrows)

**Fig. 3:**
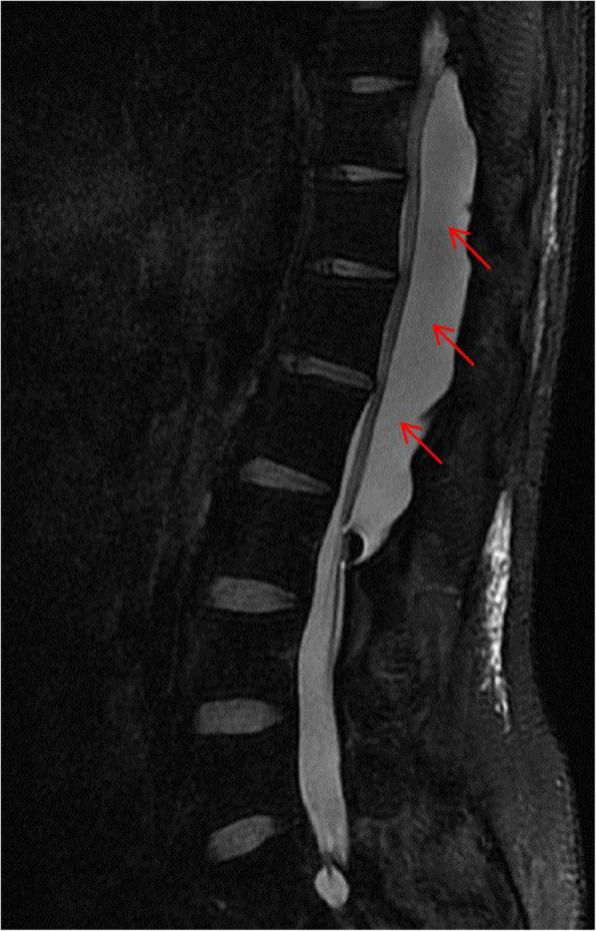
The magnetic resonance imaging (Sagittal T2-weighted) after 20 mL of fluid was aspirated from the cyst (red arrows)

**Fig. 4:**
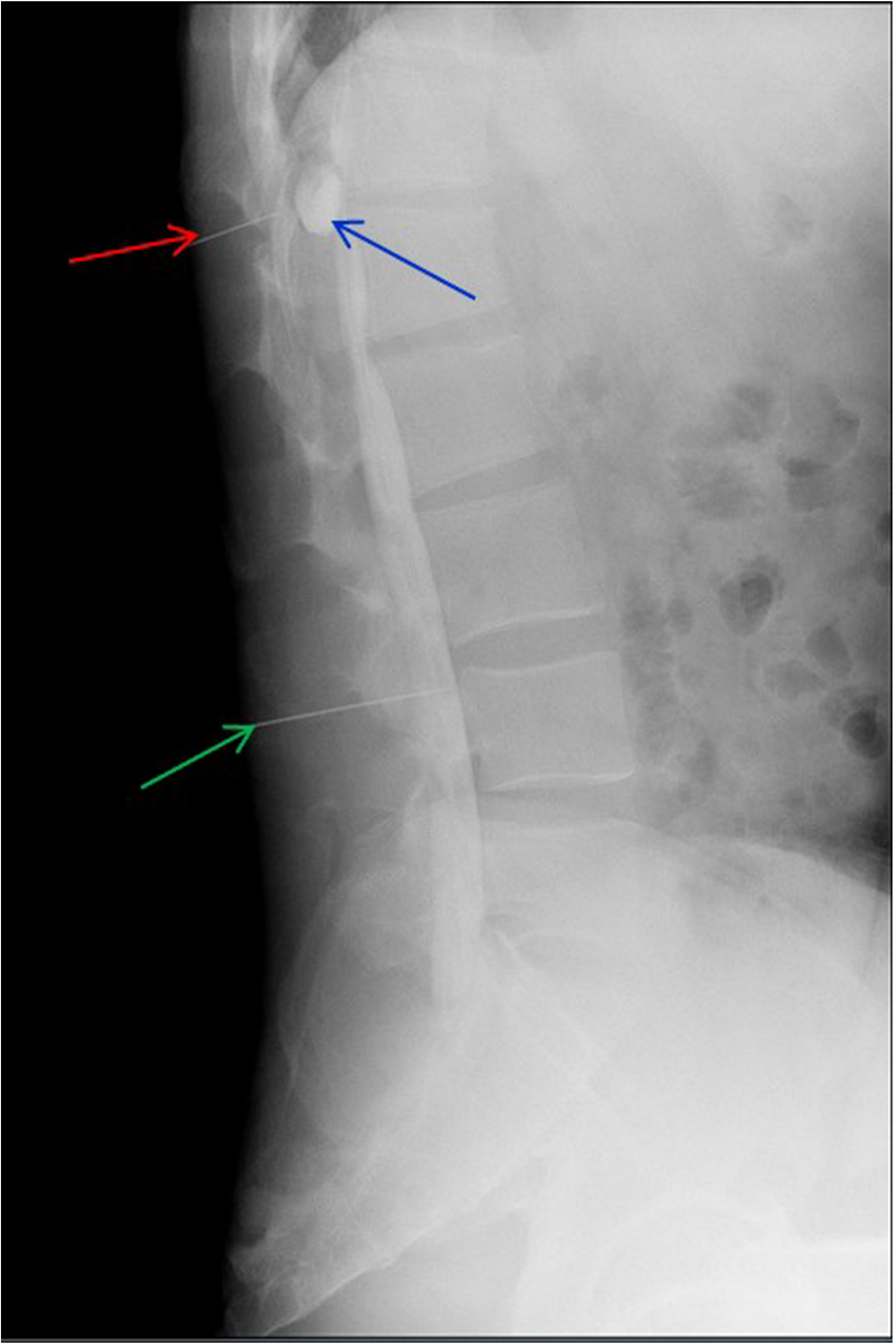
Utilizing two needles, punctured into the cystic space (red arrow) and subarachnoid space (green arrow) under digital subtraction angiography (DSA), drains the fluids. In this way, pressure is balanced between the two spaces. The 10 mL of contrast medium was then injected into the subarachnoid space (from the lower needle). Under DSA, we observed contrast medium infiltrating into the cystic space at the T12/L1 level (blue arrow)

**Fig. 5:**
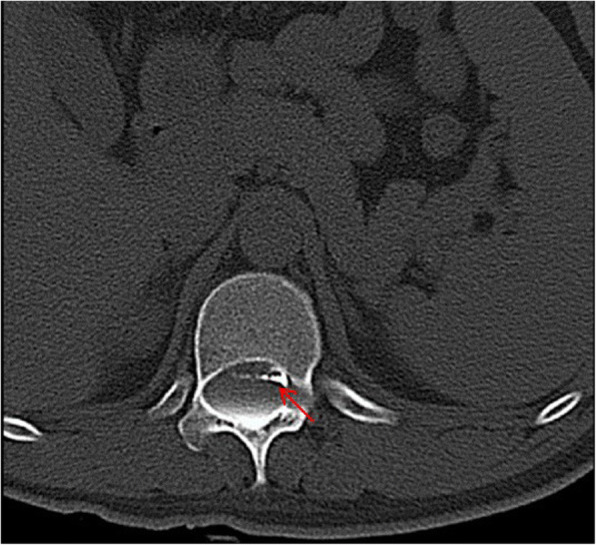
High resolution computed tomography: a funnel-shaped enhancement (red arrow) between the lower edges of the T12 body and the left nerve root

This is a 26-year-old man who experienced right lower limb weakness for 2 years and the weakness exacerbated in last half year. He visited the second affiliate hospital of the Zhejiang University School of Medicine. A physical examination indicated the result of the straight leg raising test was positive and also muscle atrophy. The muscle power of the right lower limb had decreased to grade 3. There was no sensory impairment. Magnetic resonance imaging revealed an intraspinal extradural tumor over T10 to L3 (Fig. 1). It appeared to be a spinal extradural arachnoid cyst (SEAC). To confirm whether the fistula existed between the subarachnoid space and arachnoid cyst, and to localize the position of the fistula, we performed a real-time technique. First, we injected contrast medium into the cyst under fluoroscopy. After 1 h the follow-up computed tomography (CT) was administered, and it revealed there was no contrast-infiltration into the subarachnoid space (Fig. 2). We then extracted about 20 mL of fluid from the cyst. The follow-up magnetic resonance imaging on the same day indicated the cyst did not become smaller (Fig. 3). A “one-way valve” fistula was suspected such that cerebral spinal fluid could pass into the cystic space from the subarachnoid space but could not flow in the opposite manner. Therefore we designed a procedure to localize the fistula. We penetrated two needles into the cyst and subarachnoid space separately in the L3/L4 level under digital subtraction angiography (Fig. 4). Pending the fluid drained through those two needles, we injected 10 mL of contrast medium slowly into the subarachnoid space and a little contrast medium infiltrated into the cystic space horizontally at the T12/L1 level. We then administered high resolution computed tomography (HRCT) immediately to confirm the position in the axial plane. The HRCT revealed a funnel-shaped enhancement between the lower edges of the T12 body and the left nerve root, and this is the accurate position of the fistula (Fig. 5).

### Operative method

**Fig. 6:**
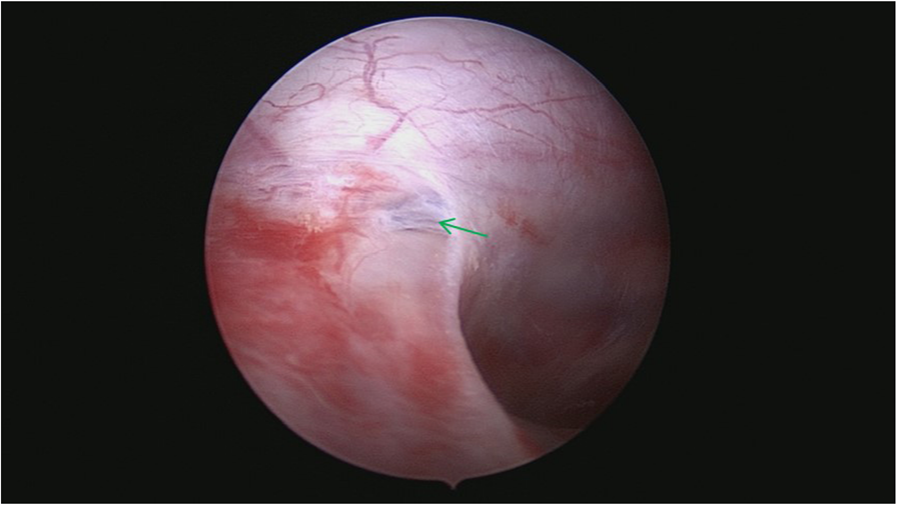
The fistula (green arrow) was discovered by spinal endoscope

**Fig. 7:**
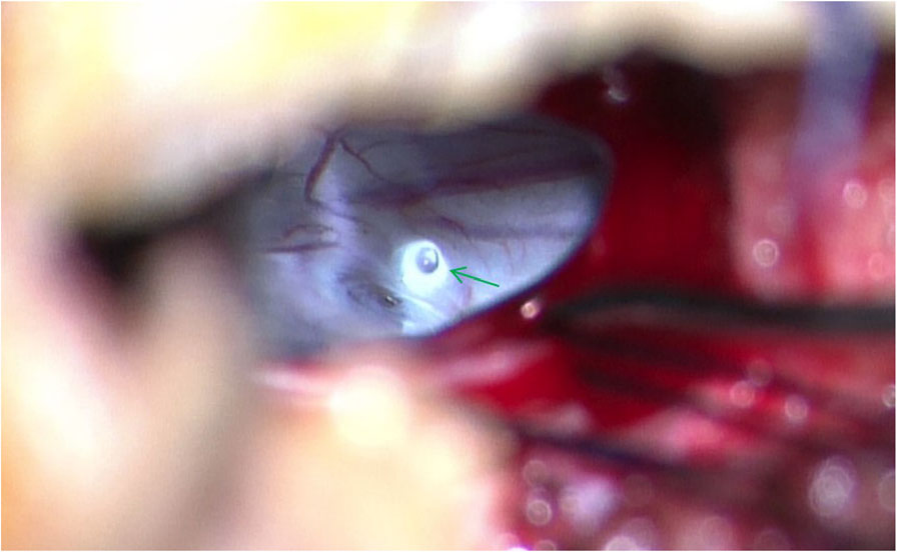
A microscopic view of the fistula leaking cerebrospinal fluid (green arrow)

**Fig. 8:**
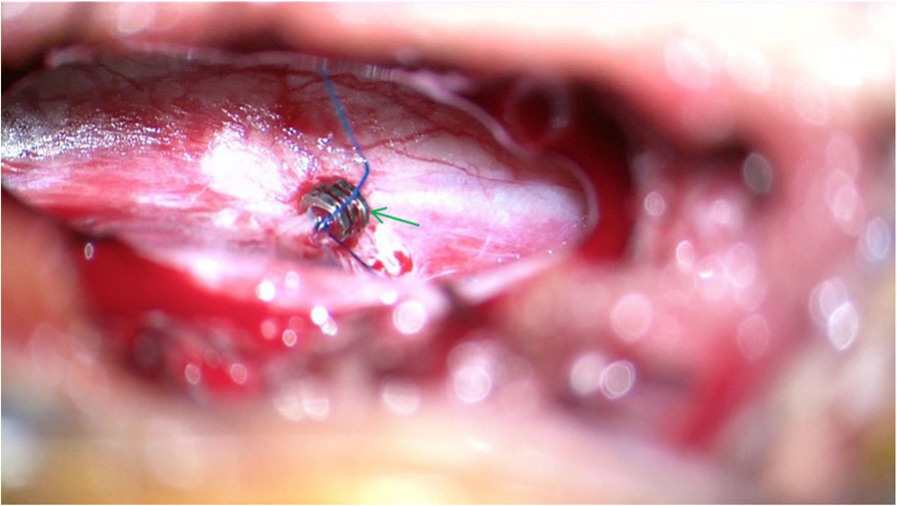
The fistula was ligated with a 7–0 Vicryl purse string suture, and an Anastoclip (green arrow) was then administered to close the fistula

**Fig. 9:**
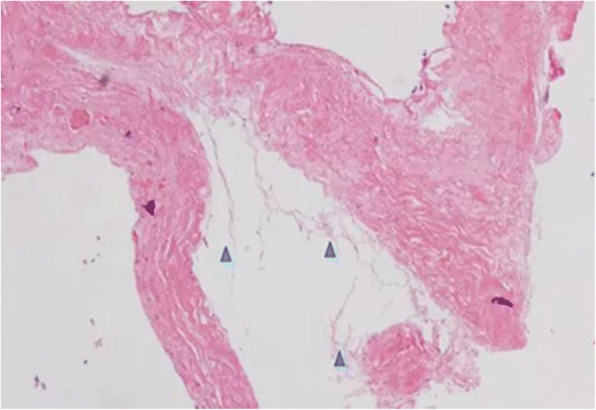
Photomicrograph: cyst wall has delicate fibrous connective tissue with calcification and loosening arachnoid tissue over inner membrane (blue arrows)

After general anesthesia and endotracheal intubation, the patient was placed in prone position. A fluoroscopy was used to determine the correct operative level, and a longitudinal incision was made 5 cm off midline at the T12 level. Muscle was dissected layer by layer, and a spinal process and left lamina were exposed. The left lamina was partially ground by cutting burr and then removed along with ligamentum flavum by Kerrison punch. After that, the cyst was exposed. Under a microscope, the cystic wall was fenestrated and it then drained off clear cystic fluid (cerebral spinal fluid). After partial excision of the cyst and evacuation of cystic fluid, a spinal endoscope (SPINENDOS, Germany) was maneuvered into the space and the fistula was detected (Figs. 6, 7). The fistula was detached from the arachnoid membrane and was ligated with a 7–0 Vicryl purse string suture. Regional leakage from the repaired site was noted. An anastoclip was then used to close the fistula (Fig. 8). Pulmonary pressure was elevated by ventilator (valsalva maneuver) to check the leakage and there was no more leakage. Hemostatic matrix and gel [Fibrin sealant kit (human), (Shanghai RAAS Blood Products Co, Ltd., Shanghai, China)] were utilized at the local region. The postoperative diagnosis was a thoracolumbar extradural arachnoid cyst. The pathological report revealed an arachnoid cyst (Fig. 9). The symptoms improved on postoperative day 2. His lower limbs regained strength with limited dorsiflexion of the right foot.

### Follow-up

**Fig. 10:**
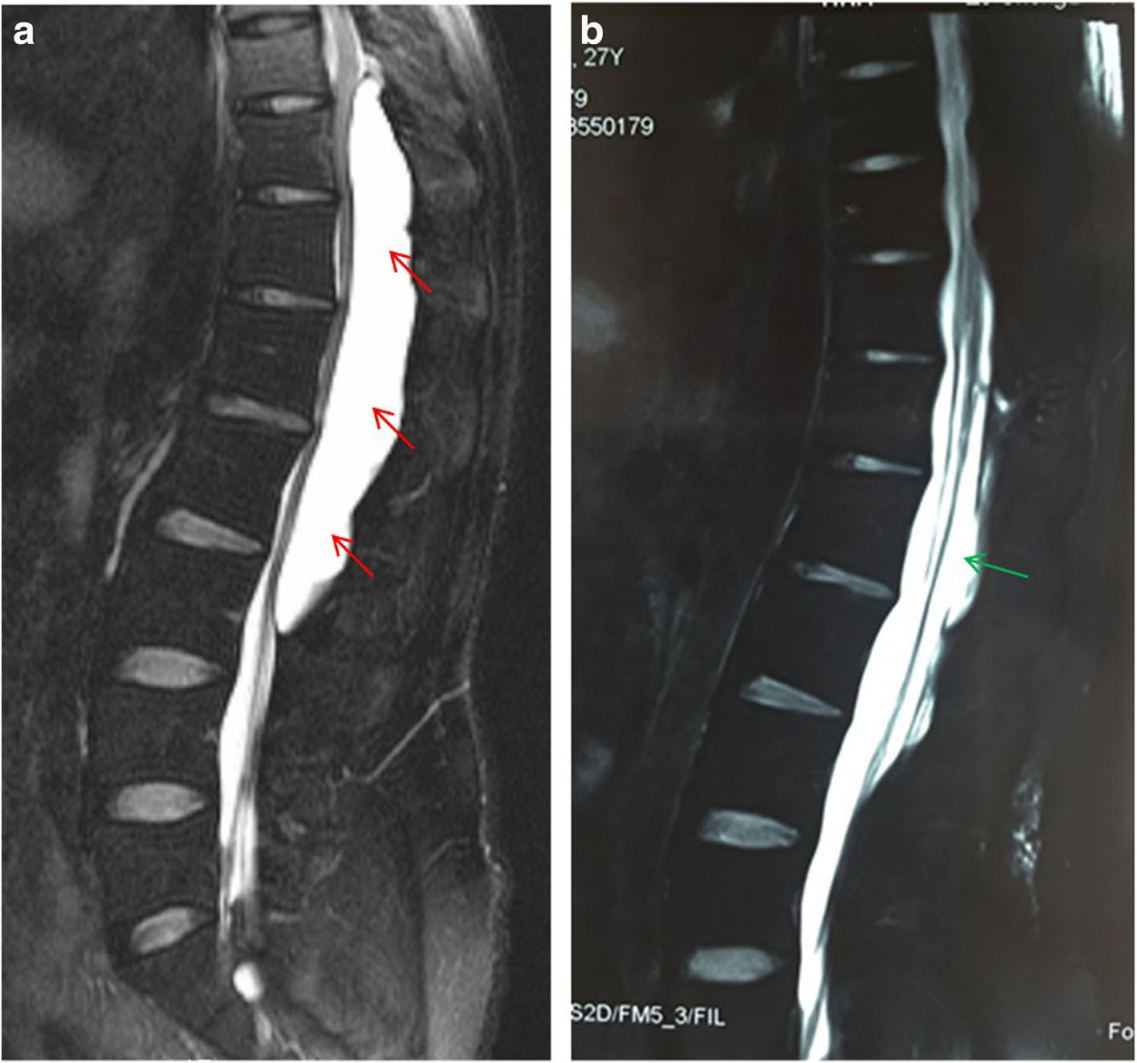
**a** The pre-operative magnetic resonance imaging (red arrows); **b** The 3-month follow-up magnetic resonance imaging: there was no recurrence of the cyst (green arrows)

Through the 3-month outpatient department follow-up, there was no more numbness or weakness of his right lower limb. The motion of dorsiflexion also improved. The 3-month follow-up magnetic resonance imaging revealed no recurrence of the previous lesion and no spinal cord compression (Fig. 10).

## Discussion and conclusions

**Fig. 11:**
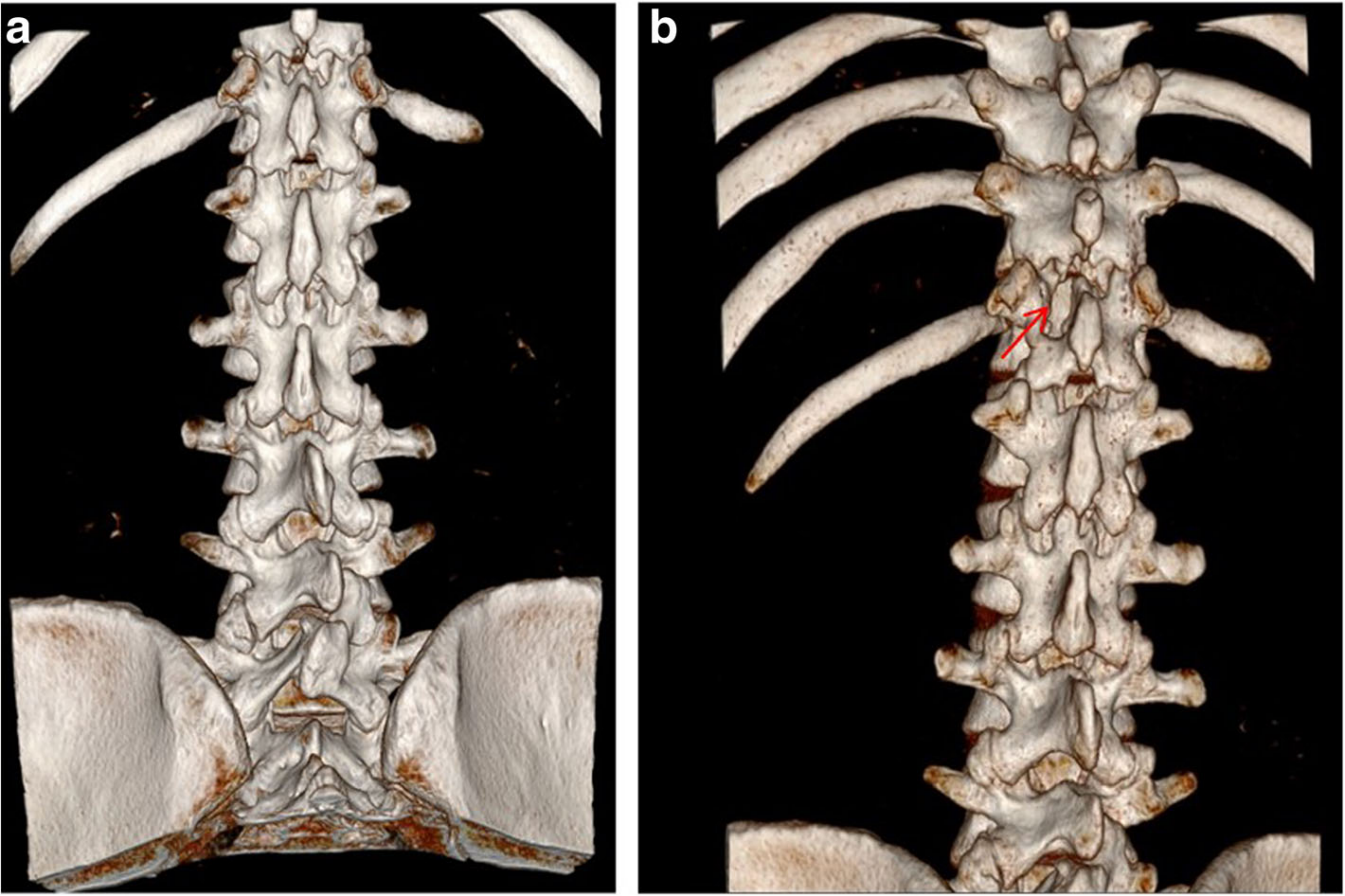
**a** The pre-operative 3-D reconstructive computed tomography (3-D RCT); **b** The post-operative follow-up 3-D RCT showing a small defect of right lamina (red arrow)

Spinal extradural arachnoid cysts are rare, and their origin and pathogenesis remain unknown. Some consider that an idiopathic spinal extradural arachnoid cyst is caused by herniation of arachnoid tissue through a congenital dural defect or invasion of a congenital dural diverticulum [1, 2]. Previous researches hypothesized that dural defects are like “one-way valves”, permitting CSF flow into the cyst under high CSF pressure from the subarachnoid space [3]. There are many surgical options including simple osseous decompression, incision and drainage, subtotal or total cyst resection, and marsupialization of the cyst [4]. Eroglu et al. reviewed 13 patients with spinal arachnoid cysts (SAC) who underwent surgical intervention. All performed fenestration of the cyst wall into the subarachnoid space at least including five total resection. There was recurrence in those patient during mean 55 months follow-up [5]. Fam et al. published 22 adults with spinal arachnoid cysts (SAC) in a single center including the six patients with extradural SAC and concluded that surgical exploration and complete resection is the treatment of choice [6]. Complete excision of the cyst followed by tight closure of the fistula by suture is suggested [1]. Total resection could be hard because the cyst might adhere tightly to the dura or the nerve tissue, or because of intraoperative bleeding from well-developed epidural venous plexuses. Intraoperatively letting a small part of the cyst wall remain should not increase recurrence since re-accumulation of fluid and recurrence of symptoms after the initial cyst removal are rare [7]. Complete cyst excision with fistula closure is the most successful surgical approach. If it cannot be achieved, either complete cyst excision without fistula closure or fistula closure with partial cyst excision can also achieve satisfactory surgical outcomes [8]. Several minimally invasive alternative surgical procedures have been reported such as selective laminectomy with fistula closure, shunts, and imaging-guided aspiration. Other techniques have been used in treating giant arachnoid cysts. Neo et al. proposed a case with extradural arachnoid cyst detected by a pulsating flow voiding observed at Cine-MRI and detected the location of the communication site of level L1 [9]. Nakagawa et al. presented a case with large extradural SAC and found the benefit at 3D constructive interference in steady state (CISS) MRI because of the additional surgery aimed at closing the dural defect [10]. Mishra et al. demonstrated intraoperative dynamic magnetic resonance myelogram for detecting the site of the fistula [11]. Gu et al. used digital subtraction cystography to detect a communicating hole between a cyst and the subarachnoid space [12], and Takamiya et al. injected pyoktanin blue into spinal arachnoid cysts to aid in total excision [13]. Endo et al. offered the less invasive surgical method of endoscopic fenestration to manage spinal intradural arachnoid cysts [14]. In this case, the giant extradural arachnoid cyst had multiple spinal segment involvement; therefore, a laminotomy or laminectomy might be performed at the multiple involving segments. The postoperative bony defect may induce structural instability and deformity, and spinal fusion and fixation should also be performed after cyst excision. Considering the young age and postoperative life quality of the patient, minimal invasive surgery was preferred and how to localize the fistula became the top priority. We proposed a preoperative method that utilizes real-time radiography under digital subtraction angiography and high resolution computed tomography. If this method accurately locates the fistula, ligation can be successful under minimally invasive laminotomy. In addition, the intraoperative use of the spinal endoscopic technique and a microscope enhances surgical safety, which decreases the damage of nerve roots and cords and other complications in the clear operative field. A postoperative follow-up 3-D reconstructive computed tomography revealed that only a small part of lamina was removed (Fig. 11). Such a small wound was one reason for the rapid postoperative recovery. A disadvantage for patients might be the radiation exposure. The follow-up period was short, so the recurrence may be adjusted in the future. This is one of our study limitations.

When managing a giant spinal extradural arachnoid cyst, accurate localization of the fistula gives assurance that minimally invasive surgery can be performed. We propose a brand new technique to localize the fistula of a spinal arachnoid cyst. The intraoperative use of a microscope and an endoscope also contributes to surgical safety. However, we need more cases with varying conditions to further evaluate this technique.

## Abbreviations

CSF: Cerebrospinal fluid
CT: Computed tomography
DSA: Digital subtraction angiography
HRCT: High resolution computed tomography
SEAC: Spinal extradural arachnoid cyst
